# Photo-Initiated Main-Chain Scission of Poly(methyl methacrylate) in Solution at Room Temperature

**DOI:** 10.3390/polym18162012

**Published:** 2026-08-18

**Authors:** Xiao Wang, Xiangze Meng, Zhiping Xu, Rui Yang

**Affiliations:** Department of Chemical Engineering, Tsinghua University, Beijing 100084, China; xiao-wan24@mails.tsinghua.edu.cn (X.W.); meng_xz@yeah.net (X.M.); xuzp15@163.com (Z.X.)

**Keywords:** poly(methyl methacrylate), photo-initiation, solution degradation

## Abstract

Poly(methyl methacrylate) (PMMA), as a widely used transparent polymer material, is highly stable because of its all-carbon backbone, which makes its chain cleavage under mild conditions challenging. In this work, we report a photo-initiated solution reaction that induces main-chain scission of PMMA at room temperature, leading mainly to molecular-weight reduction and oligomer formation. This method requires no catalysts and does not need pre-introduction of specific groups. The degradation mechanism proposed according to DFT calculations involves the photolysis of trichloromethane to produce phosgene, which then reacts with ester groups on the side chains of PMMA to form acyl chloride groups. These acyl chloride groups further cleave under light or heat, generating radicals that trigger β-scission of the PMMA main chain through a side-chain-initiated pathway. The degradation mechanism was demonstrated experimentally, and the extent of chain scission can be regulated by temperature, O_2_ and an alcohol stabilizer.

## 1. Introduction

Poly(methyl methacrylate) (PMMA) is a thermoplastic material with high optical transparency [1]. In addition to its exceptional light transmittance, PMMA boasts a series of advantageous properties, including lightweight [2], high-impact resistance [3], excellent dielectric and insulation properties [4], good chemical and weather resistance [5], biocompatibility [6], and outstanding processability [7]. The superior characteristics of PMMA have led to its widespread market applications [8], especially in the rapidly expanding liquid crystal display industry, which has resulted in a substantial and growing market size [9]. However, the scale of PMMA waste is equally increasing. According to European market statistics, approximately 10% of produced cast sheets, extrusion sheets, or granules PMMA (around 30,000 tons) became waste annually during the production process alone, with only about 8000 tons being recycled [10]. Due to the C-C bonds in the main chain, PMMA is quite stable [11]. Additionally, the all-carbon backbone of PMMA makes selective chain cleavage under mild solution conditions difficult, in contrast to condensation polymers that can undergo bond exchange or solvolytic cleavage more readily [12].

To achieve depolymerization or chain unzipping under mild conditions, it is often necessary to introduce reactive groups at the chain ends of PMMA. For instance, based on reversible deactivation radical polymerization (RDRP) [13], various groups like halogen atom (ATRP) [14], thiocarbonyl (RAFT) [15], alkoxyamine (NMP) [16] were introduced at the chain ends of PMMA. The bond energy of these groups is significantly lower than that of the C-C bond; thus, the temperature required for the depolymerization of PMMA obtained through RDRP methods is markedly reduced (<170 °C) [17]. Through RAFT polymerization, PMMA was introduced with thiocarbonylthio terminal groups, in which C-S bonds are easily broken by light irradiation at 100 °C, achieving catalyst-free depolymerization in solution or bulk [18]. ATRP polymerization introduced carbon-halogen bonds at the ends of PMMA. Yagci et al. [19] found that Cl-capped PMMA underwent significant depolymerization in solution with the existence of Mn_2_(CO)_10_ catalyst and visible light irradiation, and the extent of depolymerization increased with the temperature. Ouchi et al. [20] also investigated the depolymerization behavior of Cl-capped PMMA, finding that a controlled depolymerization reaction could be achieved at 100 °C in the presence of Ru(Ind)Cl(PPh_3_)_2_ catalyst in solution. The molecular weight of PMMA reduced, while the molecular weight distribution did not change obviously. They had reached a depolymerization rate of 24% after 24 h at 120 °C.

These degradation methods require the pre-introduction of labile groups at the chain ends of PMMA. Such PMMA is more likely to be “polymers designed for depolymerization”, rather than general commercial PMMA used in practical applications. Itaru et al. [21] used benzophenone derivatives as photo initiator; the triplet benzophenone derivatives generate free radicals on the main chain of PMMA through hydrogen absorption, followed by β-scission and depolymerization. The main chain fracture temperature of anionic PMMA was reduced from the traditional 270–330 °C to 140–180 °C, achieving low-temperature controllable depolymerization of PMMA. Anastasaki et al. [22] recently reported a main-chain-initiated depolymerization method for commercial PMMA under visible light. This method involves the cleavage of C-Cl bonds in chlorobenzene solvent under visible light to generate chlorine radicals in situ, which then undergo hydrogen transfer reactions with the methyl or methylene groups on the PMMA main chain to form carbon radicals, thereby initiating β-scission of the PMMA main chain. This method achieved high-efficiency depolymerization and monomer recovery of commercial PMMA at 150 °C, using dichlorobenzene as the solvent.

In this work, we present a photo-initiated solution reaction that can achieve main-chain scission of PMMA at room temperature, accompanied by partial depolymerization. Due to mild reaction conditions, PMMA mainly underwent chain scission to give lower-molecular-weight oligomeric products, with a small portion depolymerizing into monomer. Different from Itaru’s work [21] and Anastasaki’s work [22], we utilized the common solvent trichloromethane and did not add any photo initiator or photo catalyst. The free radicals were generated through a side-chain initiation pathway instead of hydrogen absorption on the main chain, and the whole reaction occurred at room temperature. The photo-initiated chain-scission mechanism was clarified, and a regulation strategy of this reaction was proposed accordingly.

## 2. Experimental

### 2.1. Materials

Commercial atactic PMMA pellets (IF850) were purchased from LG Chem. Co. Ltd. (Seoul, Republic of Korea). The PMMA exhibits an average molecular weight of ~40,000 and a polydispersity index (PDI) of ~2.0. Its melt flow index is 13 g/10 min at 230 °C with a 3.8 kg load. Thermogravimetric analysis (TGA) under nitrogen atmosphere determined its onset decomposition temperature to be more than 320 °C. Trichloromethane (TCM) with 99.9% purity HPLC grade and concentrated hydrochloric acid with AR grade (36–38 wt%) were purchased from Beijing Tongguang Fine Chemical Company (Beijing, China). Tetrahydrofuran (THF) with 99.9% purity HPLC grade was purchased from Energy Chemical (Shanghai, China). Dichloromethane (DCM) with 99.5% purity AR grade was purchased from Beijing Pure Chem. Co., Ltd. (Beijing, China). Xylene with 99% purity AR grade was purchased from J&K Scientific (Beijing, China). Methanol with 99.9% purity HPLC grade was purchased from Fisher Chemical (Waltham, MA, USA). Anhydrous ethanol with 99.7% purity AR grade and anhydrous sodium sulfate with 99.0% purity AR grade were purchased from General Reagent (Shanghai, China). Alumina with 99.0% purity AR grade was purchased from Sinopharm Chemical Reagent Co., Ltd. (Shanghai, China).

Tetrahydrofuran (THF) and trichloromethane (TCM) were purified before use. Tetrahydrofuran was purified by distillation. Trichloromethane was passed through a chromatography column filled with alumina, then mixed with water in a ratio of about 1:1 for extraction and separation, and finally added to anhydrous sodium sulfate and stirred overnight to obtain purified trichloromethane.

### 2.2. Degradation and Characterization

**Figure 1:**
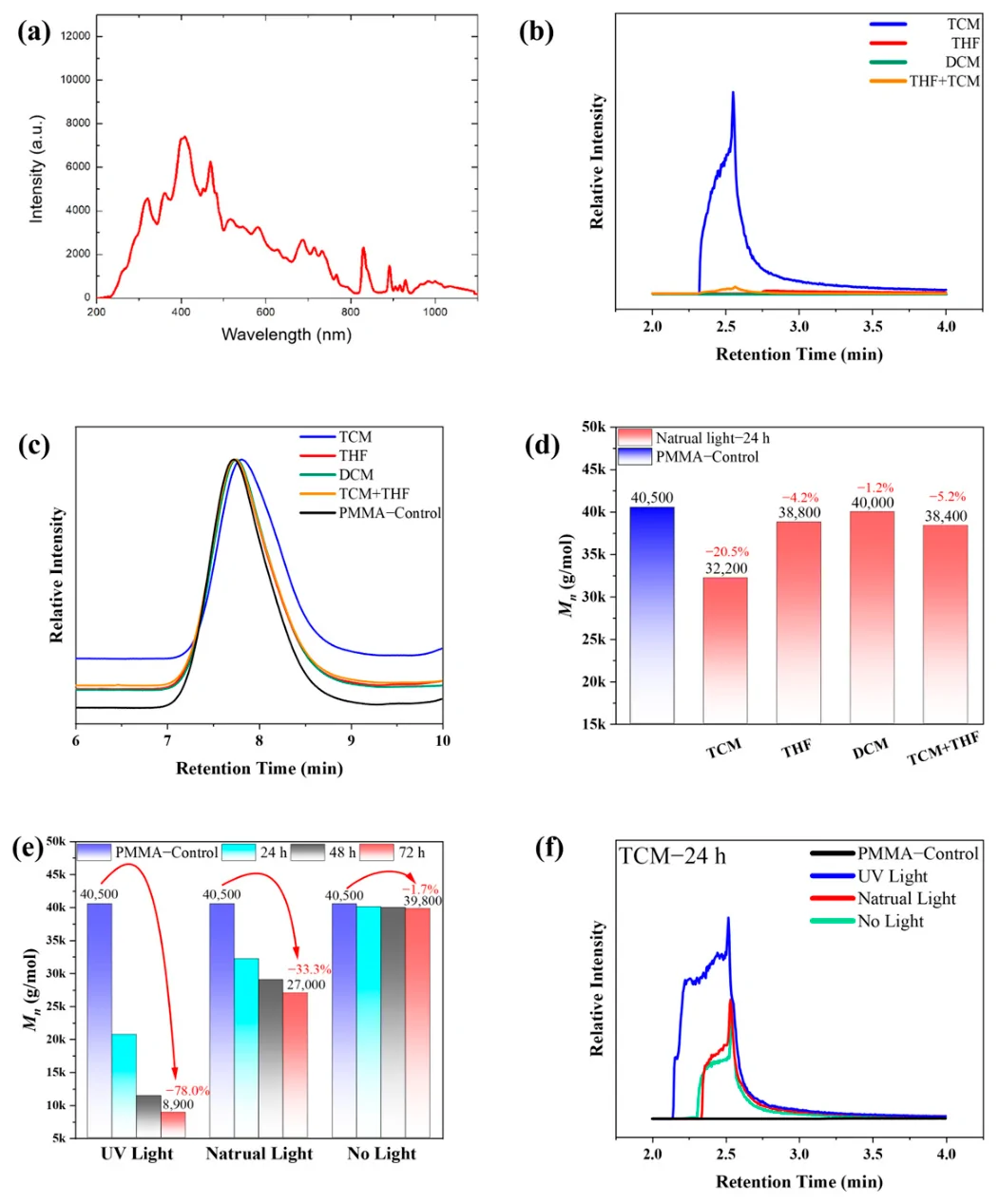
(**a**) Spectrum of xenon lamp in UV experiment; the degradation of PMMA in different solvents (TCM, THF, DCM, THF+TCM) under natural light irradiation for 24 h; (**b**) peak intensity of MMA monomer (*m*/*z* = 100) in; (**c**) GPC curves (normalized by maximum intensity); (**d**) changes in molecular weight of PMMA; the degradation reaction of PMMA in TCM under different lighting conditions; (**e**) changes in molecular weight after different reaction times; (**f**) peak intensity of MMA monomer (*m*/*z* = 100) after 24 h.

50 mg/mL PMMA solution in trichloromethane was exposed to Xenon lamp (BBZM-I, Bobei Lighting Company, Anhui, China) or natural light at various temperatures. The emission spectrum of the xenon lamp is provided in Figure 1a, and its optical power density (365 nm) is approximately 30 mW/cm^2^. Natural light includes indoor sunlight and LED light. To avoid absorption of ultraviolet light by the glass of the flask, the xenon lamp head was directly inserted into the flask, and xenon lamp head is 5 cm away from the liquid level of the solution.

The molecular weight of PMMA samples was determined using gel permeation chromatography (1260 Infinity II, Agilent Technologies, Santa Clara, CA, USA) at 30 °C, with THF as the mobile phase. The percentage of molecular weight decrease was used to characterize the degree of degradation.

Degradation products were identified using GCMS-QP2010SE gas chromatography-mass spectrometry (Shimadzu Corporation, Kyoto, Japan). 10 μL solution was injected into the pyrolyzer at 300 °C for 0.5 min to evaporate the volatile components in the solution. Volatile components were introduced into the chromatographic column using helium gas as a carrier gas for separation, and then identified by the MS detector with *m*/*z* = 33–600 amu. Peak intensity and area of MMA monomer (*m*/*z* = 100) under various degradation conditions were compared.

EPR spectra were recorded at room temperature with a JES-FA200 continuous wave electron paramagnetic resonance spectrometer from JEOL Ltd. (Tokyo, Japan). N-*tert*-Butyl-alpha-phenylnitrone (PBN) was added as the spin-trapping agent with a mass concentration of 10 mg/mL to capture carbon centered free radicals. The sample solutions were introduced into the resonator by means of a 0.3 mm inner diameter quartz capillary. The irradiation was performed with the Perfectlight PLS-SXE300+/UV xenon lamp (Beijing, China). The wavelength range is 320–780 nm and used optical filter to suppress the emission below 360 nm. All CW EPR spectra were acquired with the following spectrometer settings: microwave frequency ≈ 9.4 GHz, center field = 336.0 mT, sweep width = 10 mT, sweep time = 1.0 min, modulation amplitude = 0.2 mT, modulation frequency = 100 kHz, microwave power = 0.998 mW, conversion time = 0.1 s, and time constant = 0.03 s. Mn^2+^ marker was used as a magnetic field standard, including both horizontal-axis shifting and vertical-axis normalization.

### 2.3. DFT Calculations

All density functional theory (DFT) and time-dependent DFT (TDDFT) calculations were performed to investigate the photochemical behavior of trichloromethane, PMMA, and its acyl chloride-functionalized derivative (PMMA-Cl), where the methyl ester group (–COOCH_3_) in PMMA was replaced by an acyl chloride group (–COCl). These calculations were carried out using ORCA 6.0.

The C–Cl bond dissociation pathway in trichloromethane was mapped using relaxed potential energy surface (PES) scans at the r^2^SCAN-3c level [23]. For each scanned geometry, vertical electronic excitations were computed using TDDFT with the PBE0 hybrid functional [24] and def2-TZVPP basis set [25], incorporating spin–orbit coupling (SOC). The apparent bond dissociation energy (BDE) as a function of excitation wavelength was derived by correlating the first excited state (T_1_) transition energies with the corresponding ground state single-point energies (SPEs).

For PMMA and PMMA-Cl, geometry optimizations were first conducted for the first excited state using r^2^SCAN-3c. Subsequent geometry optimization calculations at the ground state generated relaxation pathways. Key intermediate geometries along these pathways were subjected to ground state SPE calculations at the PBE0/def2-TZVPP level, while their excitation wavelengths were computed via TDDFT. In fact, the photolysis of the C-Cl bond in a polymeric system was reported by Anastasaki et al. [22]. According to their DFT results, the molecule at the minimum point cannot be excited under the solar irradiation, though the EPR results validated the photolysis. This is because using only the minimum geometry to calculate the UV-Vis spectrum cannot reflect the real condition. At temperature above 0 K, the vibration of bonds, including the C-Cl bonds, cannot be neglected. From their calculation results, we know that a photoactivation energy ($E_{photo-act}$) is required to elongate the C-Cl bond to reduce the lowest required excitation energy into the available range of the light source provided. As a result, a lower $E_{photo-act}$ value indicates a stronger tendency for the molecule to photolyze. To provide a clear definition, in this work, the photoactivation energy was defined as the energy difference between the ground state SPE of a given configuration ($E_{GS}^{config}$) and the global S_0_ minimum energy ($E_{GS}^{min}$):

$$E_{photo-act}=E_{GS}^{config}-E_{GS}^{min}$$

This quantity represents the thermal energy required to reach specific molecular geometries capable of absorbing photons at corresponding wavelengths computed from the transition energies. The resulting $E_{photo-act}$-vs-wavelength correlation establishes the thermodynamic threshold for photoinduced reactions, where higher-energy configurations enable absorption of longer-wavelength photons.

Ab initio molecular dynamics (AIMD) simulations were performed on the first excited state at 500 K using r^2^SCAN-3c. The time step was 1 fs. Bond dissociation kinetics were evaluated by monitoring bond lengths in PMMA and PMMA-Cl.

The energies of PMMA and PMMA-Cl reactions at the first excited state were calculated at the PBE0/def2-TZVPP level on r^2^SCAN-3c-optimized geometries. The stepwise energy diagram for radical generation was constructed by comparing the SPEs of parent molecules, transition states (where applicable), and corresponding radical products.

## 3. Result and Discussion

### 3.1. Photo-Initiated Main-Chain Scission of PMMA in Trichloromethane (TCM)

Figure 1 shows the reaction results of PMMA in different solvents after 24 h at room temperature under natural light conditions. MMA peak intensity in different solvents was compared in Figure 1b as auxiliary evidence for the formation of low-molecular-weight products. From the peak area of MMA monomer, it can be observed that a distinct MMA monomer peak appeared in trichloromethane (TCM) solvent. However, no significant signal of MMA monomer was observed in other solvents. Figure 1c shows the molecular weight changes. In TCM solvent, the GPC curve shows an obvious shift towards lower molecular weight, with a decrease of approximately 20.5% in molecular weight (Figure 1d). Py-GC/MS and GPC results together indicate that PMMA undergoes catalyst-free molecular-weight reduction in TCM solution under room-temperature natural-light irradiation, with main-chain scission as the dominant outcome.

Further, we studied the degradation behavior of PMMA solution in TCM in dark (no light), under natural light and UV light conditions. Figure 1e shows the changes in the molecular weight of PMMA after 24, 48, and 72 h. The degradation degree increased with time, and was significantly enhanced by UV light. The molecular weight of PMMA decreased by 78% after 72 h under UV light. Almost no degradation occurred in dark. This indicated that light is a necessary triggering condition for the degradation of PMMA in TCM. Figure 1f shows the peak intensity of MMA monomer after 24 h. Previous experimental results have proved that PMMA does not undergo degradation in DCM (Figure 1b,d); we dissolved PMMA in DCM at the same concentration and directly subjected it to the Py-GC/MS test as a control. There is a maximal MMA monomer concentration under UV light. It is interesting that the peak intensity of MMA monomer in dark is close to that under natural light (Figure 1f), although there are nearly no molecular weight changes in dark (Figure 1e). This contradiction indicated that high-temperature flash evaporation during Py-GC/MS testing might also cause the degradation of PMMA in TCM, which will be demonstrated in Section 3.3.

### 3.2. Degradation Mechanism of PMMA in TCM

**Figure 2:**
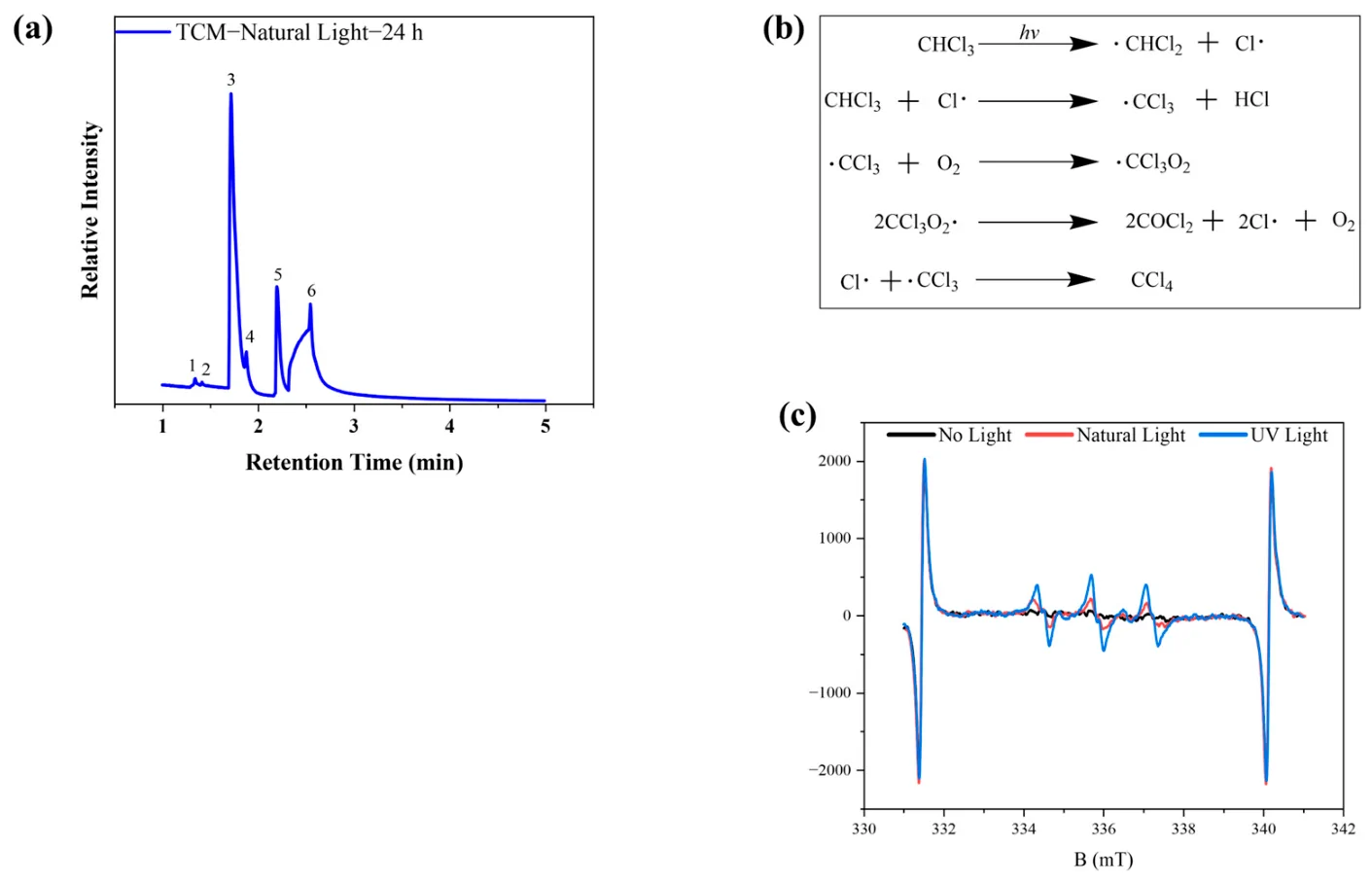
(**a**) Total ion chromatogram of volatile components in TCM after 24 h of natural light exposure; (**b**) photooxidation mechanism of trichloromethane to obtain phosgene and tetrachloromethane with permission of Reference [27]; (**c**) EPR spectra of pure TCM in dark, under natural light and UV light, with PBN as the spin-trapping agent for carbon centered free radicals.

To reveal the PMMA degradation mechanism in TCM, the volatile components in PMMA solution were analyzed first. Figure 2a shows the total ion chromatogram of PMMA solution after 24 h under natural light exposure in TCM. The volatile component identification is listed in Table 1. Due to the volume of the pyrolyzer, the carrier gas was unable to fully introduce a large amount of solvent into the gas chromatography simultaneously, resulting in a bimodal appearance of the solvent TCM. Among all the volatile components, we found the presence of phosgene and tetrachloromethane, which should be photooxidation products of trichloromethane. The photocatalytic oxidation of trichloromethane involves a series of free radical reactions, resulting in a series of chlorinated alkanes [26]. The mechanism for generating phosgene and tetrachloromethane is shown in Figure 2b [27]. Figure 2c shows the EPR spectra of pure TCM in dark, under natural light and UV light. There are obvious signals of free radicals under natural light and UV light and no signal in dark, which further prove the photolysis mechanism in Figure 2b. Akihiko Tsuda et al. [28] found that the photocatalytic oxidation of TCM can occur under white LED (400–750 nm light) if a catalytic amount of Cl_2_ is added to O_2_ gas. Therefore, we confirmed that the photocatalytic reaction of TCM occurred under our experimental conditions and generated phosgene. Combining with the analysis of volatile components of PMMA solutions, we have also verified the photocatalytic mechanism of TCM to obtain phosgene and tetrachloromethane. Phosgene has strong reactivity [29] and should play a crucial role in this degradation process.

The photo-initiated chain-scission mechanism of PMMA in TCM was investigated by DFT simulations. Firstly, the photolysis of trichloromethane generating reactive chlorine species was confirmed. Subsequently, calculations supported that the UV-Vis absorption was enhanced by the formation of acyl chloride groups on PMMA. Further calculations revealed reduced energy barriers for side-group dissociation in acyl chloride-modified structures compared to pristine PMMA. Finally, the radical reaction pathway leading to main-chain β-scission was mapped through transition-state analyses, demonstrating the synergistic photochemical cascade from initial chloroform activation to final chain scission events.

**Figure 3:**
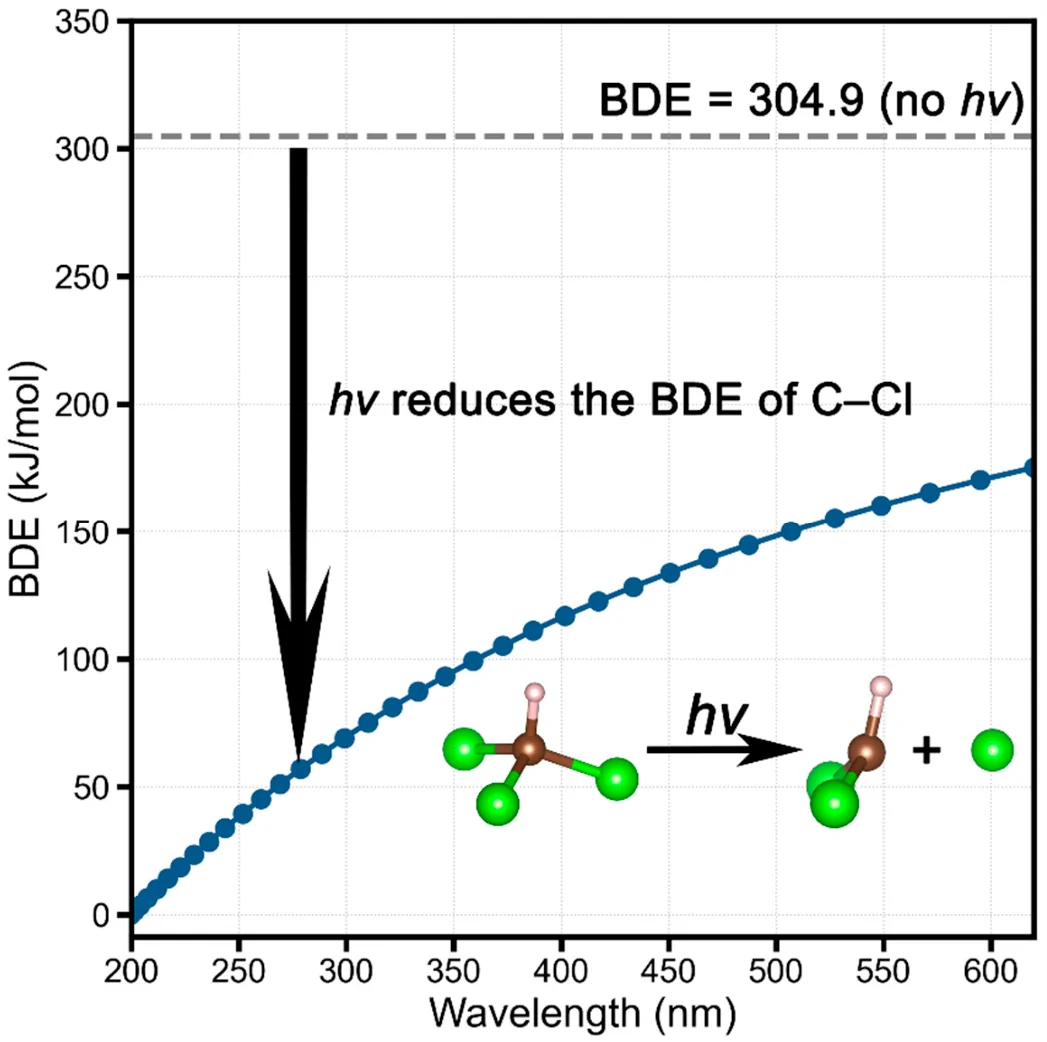
Apparent bond dissociation energy (BDE) of the C–Cl bond in trichloromethane as a function of excitation wavelength. The apparent BDE is defined as the ground-state BDE minus the photon energy (hν).

Figure 3 demonstrates the wavelength-dependent reduction in apparent C-Cl bond energy (BDE) for trichloromethane under UV-Vis irradiation, consistent with the proposed photolysis mechanism in Figure 2b. In the absence of light, the bond energy remains at 304.9 kJ/mol. Under photoirradiation, BDE decreases sharply, reaching values below 100 kJ/mol at wavelengths <350 nm. The underlying mechanism for this wavelength-dependent BDE reduction can be rationalized from the electronic structure of the C–Cl bond. Upon photoexcitation, an electron is promoted from the C–Cl σ-bonding orbital to the σ* antibonding orbital. Population of the σ* orbital effectively cancels the bonding interaction between carbon and chlorine, leading to a drastic decrease in bond order and, consequently, a strong weakening of the C–Cl bond. At shorter wavelengths, the higher photon energy accesses excited states that lie further up on the potential energy surface, where the bond is more elongated and the barrier to dissociation becomes substantially reduced. This σ → σ* photodissociation behavior is well documented for alkyl halides and provides a consistent theoretical basis for the observed trend in Figure 3. This phenomenon aligns with the mechanism reported by Anastasaki et al. [22], where C-Cl bond elongation under photoexcitation reduces the HOMO-LUMO gap, facilitating electron transfer to antibonding orbitals. The shortened photon penetration depth at shorter wavelengths (<350 nm) enhances this bond-weakening effect, as higher-energy photons promote greater electron structural distortion and orbital reconfiguration. It should be emphasized that the BDE values plotted in Figure 3 are apparent bond dissociation energies, rather than the intrinsic ground-state BDE. The apparent BDE is defined as the ground-state C–Cl bond dissociation energy (304.9 kJ/mol) minus the photon energy (hν) BDE_apparent_ = BDE_ground_ − hν. When this apparent value approaches zero, it indicates that the photon energy is thermodynamically sufficient to reach the dissociation threshold, but this does not imply a barrierless process under ambient conditions. This treatment nevertheless provides a qualitative trend that shorter-wavelength irradiation increasingly reduces the external thermal energy required to cleave the C–Cl bond.

**Figure 4:**
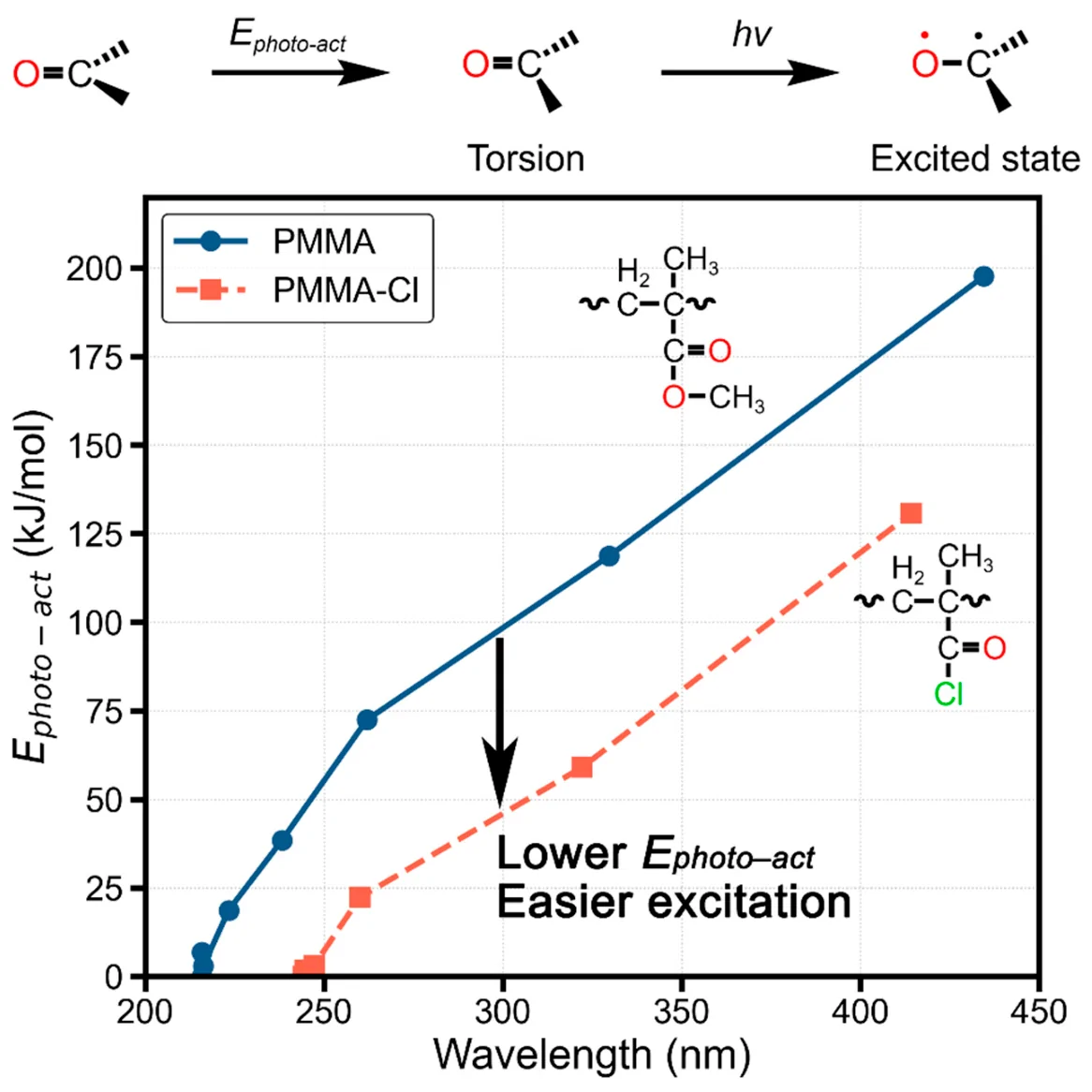
Calculated photo-activation energy ($E_{photo-act}$) of pristine PMMA and its acyl chloride-functionalized derivative (PMMA-Cl) as a function of excitation wavelength. Lower $E_{photo-act}$ values indicate a smaller thermal barrier to reach a photoactive geometry.

Before discussing the trend in Figure 4, it is necessary to clarify the physical meaning of the photo-activation energy ($E_{photo-act}$). Unlike the photon energy itself, $E_{photo-act}$ is defined as the thermal energy required for the molecule to adopt a specific nuclear configuration (primarily via C–Cl bond elongation) at the ground state, so that its vertical excitation energy becomes resonant with the incoming photon. In other words, a lower $E_{photo-act}$ value indicates that the C–Cl bond can be photolyzed with less thermal assistance. As shown in Figure 4, PMMA-Cl exhibits systematically lower $E_{photo-act}$ values than pristine PMMA across the entire wavelength range, with an average reduction of approximately 50 kJ/mol. This marked difference arises from the introduction of the acyl chloride group. The electron-withdrawing nature of the –COCl moiety, combined with the heavy-atom effect of chlorine, lowers the LUMO energy level and reduces the HOMO–LUMO gap. Consequently, the photoactive C–Cl bond in PMMA-Cl becomes significantly more susceptible to cleavage upon photoexcitation, requiring substantially less geometric distortion compared to the stable ester groups in pristine PMMA. This trend provides strong theoretical support for the proposed side-chain-initiated pathway, where acyl chloride groups act as the primary light-sensitive trigger. Figure 4 compares the wavelength-dependent photoactivation energy ($E_{photo-act}$) for PMMA and its acyl chloride-functionalized derivative (PMMA-Cl). Across all tested wavelengths, PMMA-Cl exhibits systematically lower $E_{photo-act}$ values compared to pristine PMMA, with an average reduction of approximately 50 kJ/mol. For instance, at 300 nm irradiation, $E_{photo-act}$ decreases from around 100 kJ/mol (PMMA) to around 50 kJ/mol (PMMA-Cl), consistent with trends observed at other wavelengths.

**Figure 5:**
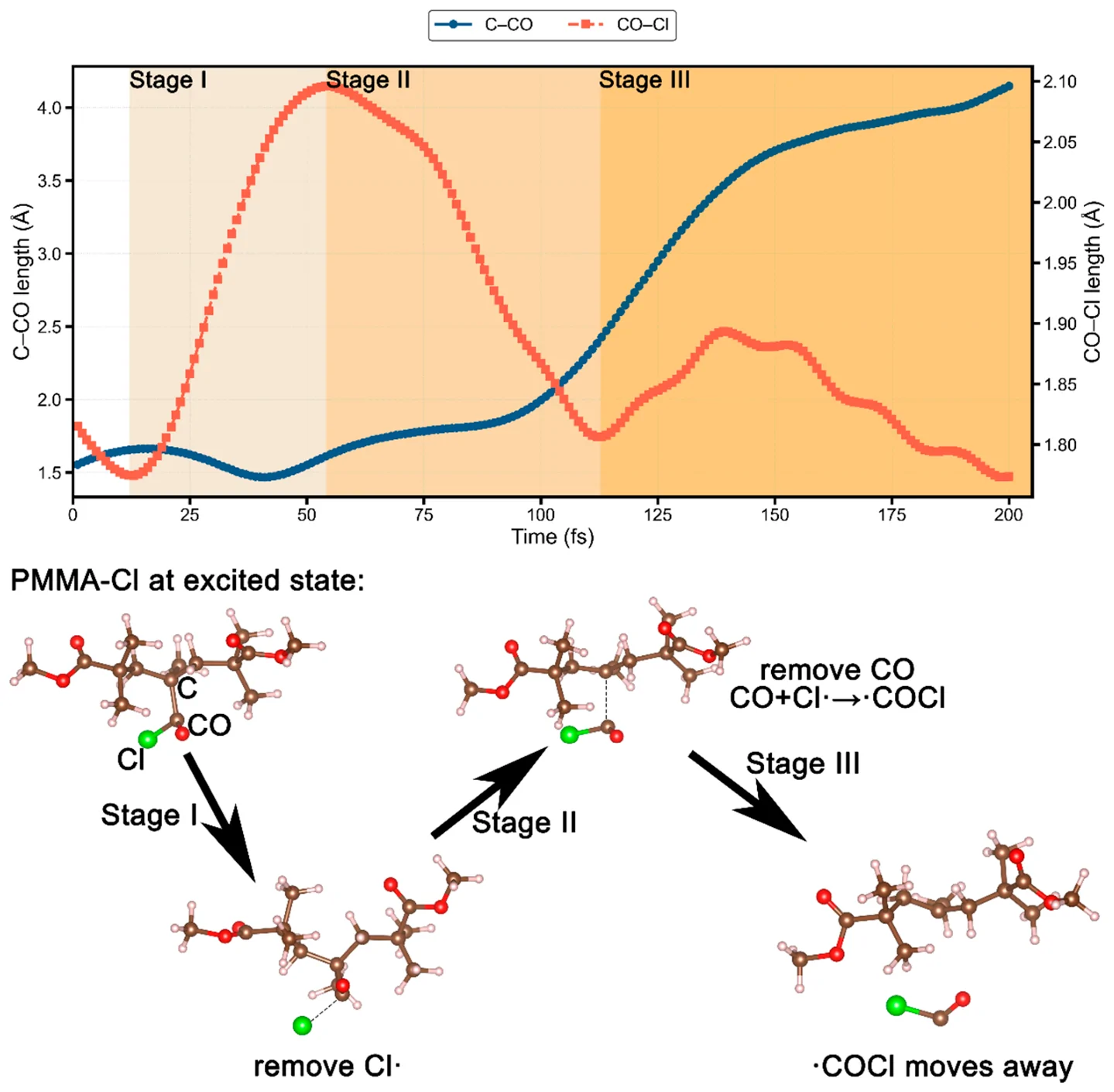
AIMD of PMMA-Cl at excited state. The oscillatory behavior after 110 fs reflects residual inter-fragment interactions prior to full separation.

It should be noted that the AIMD simulations were performed at an elevated temperature of 500 K. This choice was made purely as a computational strategy to accelerate bond dissociation events within the limited femtosecond-to-picosecond timescale accessible to AIMD. At room temperature (ca. 298 K), thermal fluctuations are insufficient to drive C–Cl bond cleavage on such a short timescale. The 500 K simulation does not aim to replicate the experimental conditions, but rather to enhance sampling along the reaction coordinate and thus reveal the preferred dissociation pathway. This accelerated dynamics approach is widely employed in computational chemistry to probe reaction mechanisms in rare-event systems. According to the AIMD results of PMMA-Cl in the excited state, the -COCl group was vulnerable to dissociation from the PMMA chain (Figure 5). The dissociation of the -COCl group occurs in three stages. In Stage I, the Cl radical dissociates, accompanied by elongation of the CO-Cl bond while the C-CO bond remains nearly unchanged (10–55 fs). In Stage II, CO dissociation followed by Cl capture forms a COCl· radical, characterized by contraction of the CO-Cl bond and concurrent elongation of the C-CO bond (55–110 fs). In stage III, further dissociation of -COCl proceeds with continuous lengthening of the C-CO distance and oscillatory variations in the CO-Cl bond length (>110 fs). The non-periodic oscillations in the CO–Cl bond length after 110 fs are attributed to the residual interactions between the Cl· radical and the CO fragment before their complete spatial separation. Within the finite simulation temperature, the two fragments remain in a confined region due to weak attractive forces, leading to the observed damped oscillatory motion. This feature is characteristic of a stepwise dissociation process within the femtosecond timescale.

**Figure 6:**
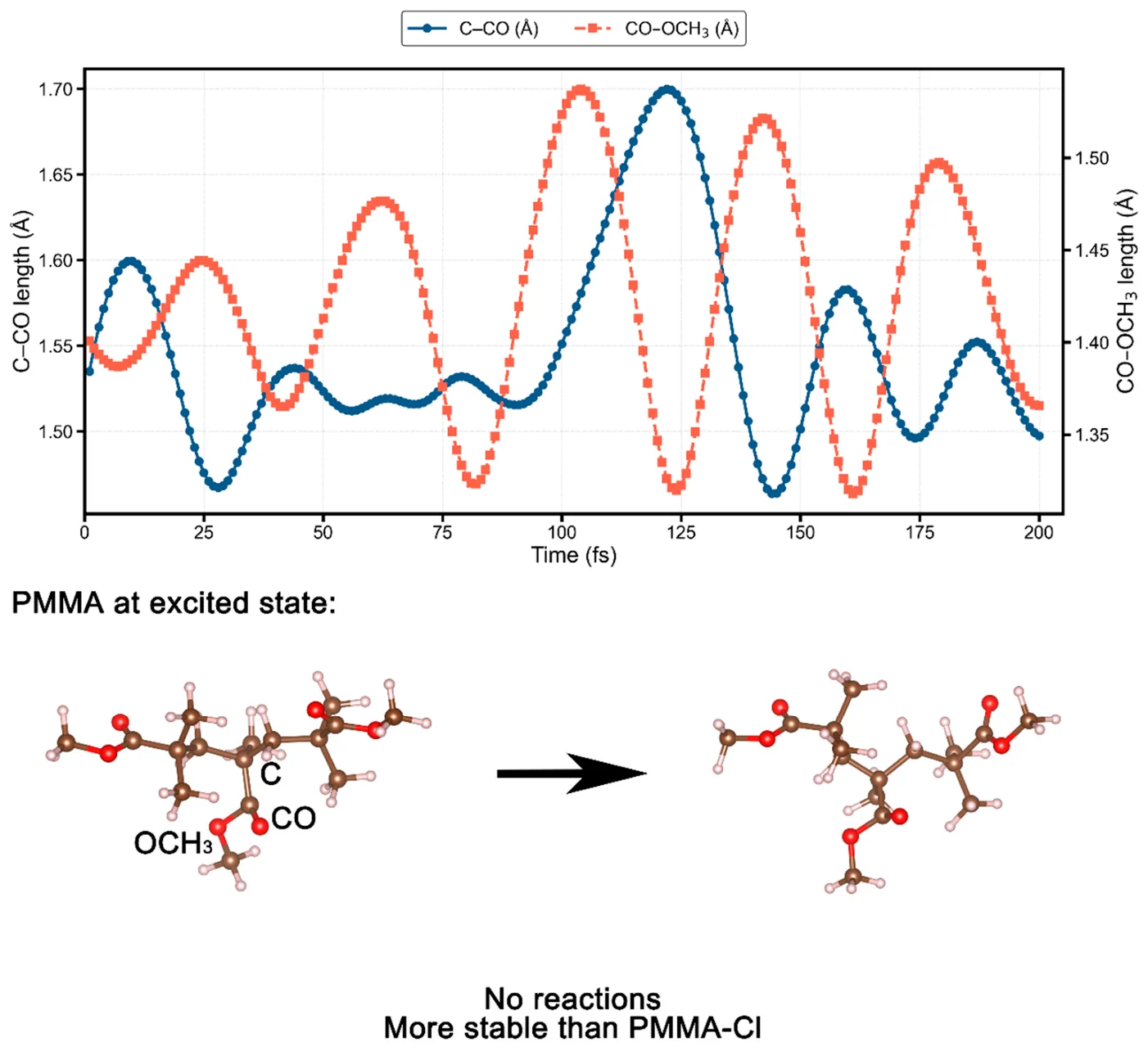
AIMD of PMMA at T_1_ state.

In contrast, excited-state pristine PMMA exhibited no reactive behavior under identical AIMD conditions, maintaining structural integrity throughout the simulation. All bonds remained stable, as exemplified by the C-CO and CO-OCH_3_ linkages monitored in Figure 6, which displayed only minor periodic fluctuations without any detectable bond cleavage events. These minor fluctuations are simply the thermal vibrational motions of the atoms at the finite simulation temperature, with all bonds oscillating around their equilibrium positions. This pronounced stability contrasts sharply with the progressive dissociation patterns observed in chlorinated PMMA derivatives, highlighting the critical role of acyl chloride formation in enabling photodegradation pathways.

**Figure 7:**
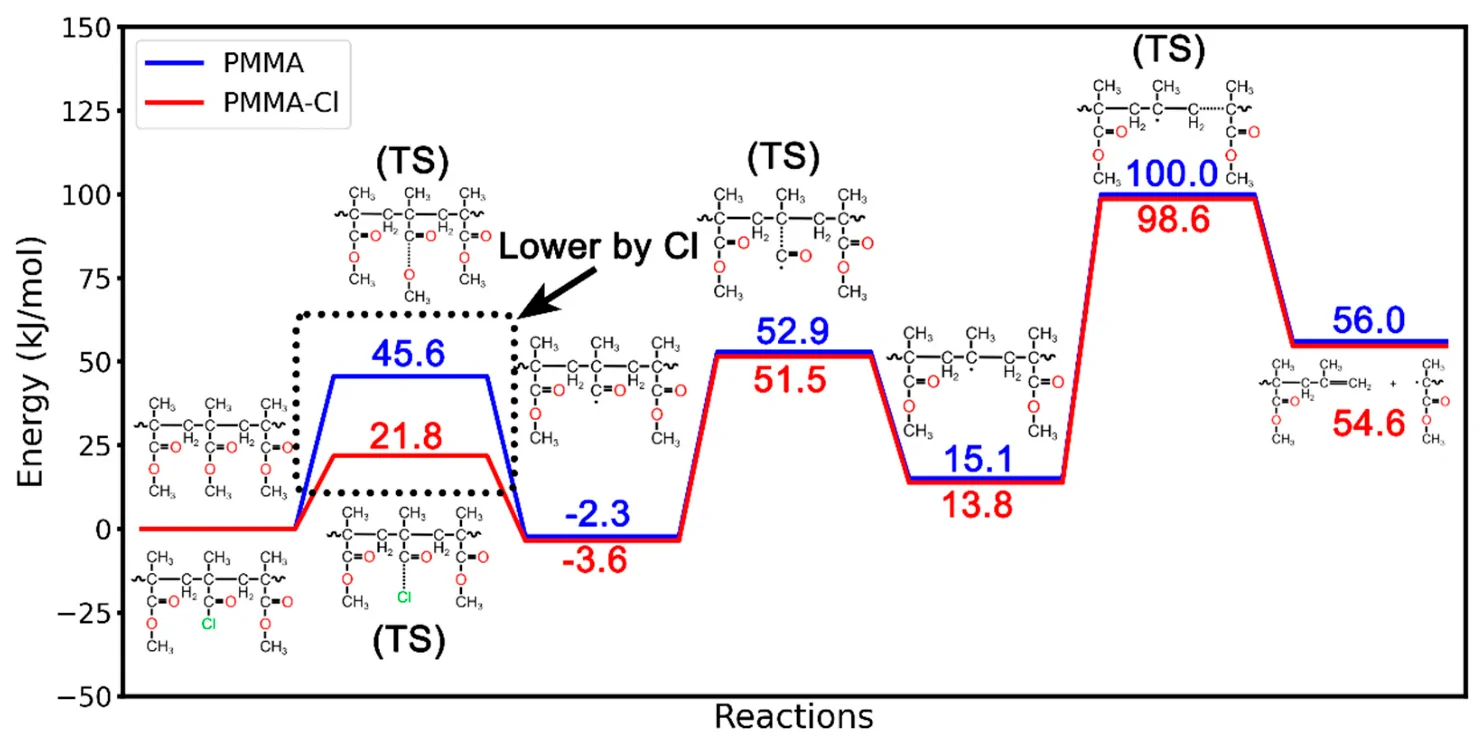
Stepwise SPE diagram of the reactions of PMMA and PMMA−Cl in the T_1_ state.

Figure 7 shows the calculated stepwise single-point energy (SPE) diagram of the reactions of PMMA and PMMA-Cl in the excited state. Replacement of -COOCH_3_ with -COCl induces a pronounced reduction in the first activation barrier from 45.6 to 21.8 kJ/mol, while subsequent reaction steps maintain nearly identical energy profiles (Figure 7). This selective barrier-lowering effect demonstrates that chlorination primarily alters reaction kinetics rather than thermodynamic driving forces.

**Figure 8:**
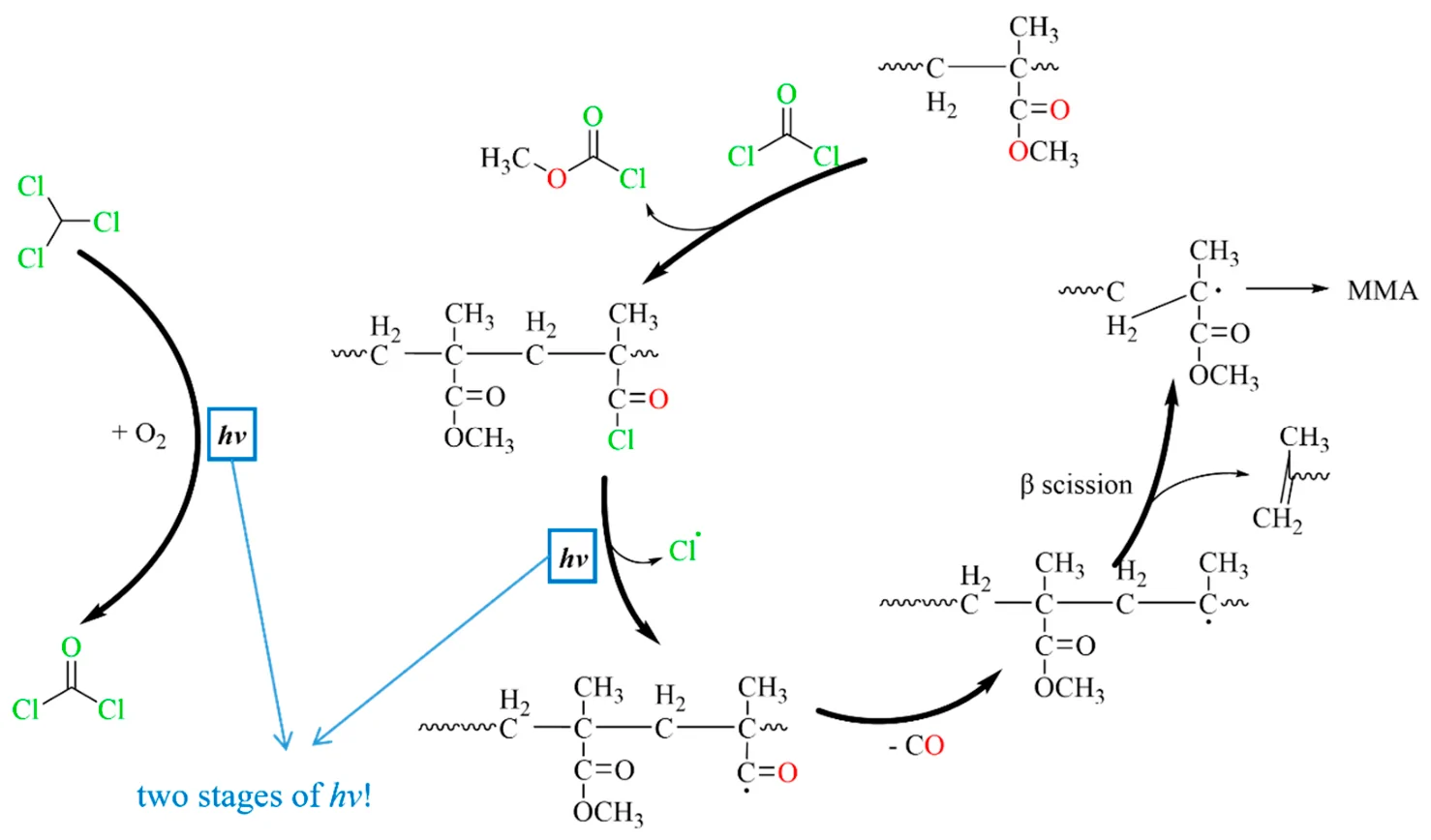
Photo-initiated degradation mechanism of PMMA in TCM.

By analyzing the reaction products and conducting DFT simulations, we proposed a side-chain-initiated main-chain scission mechanism as shown in Figure 8. Once the photooxidation of trichloromethane occurs, the product phosgene will react with ester groups on the side chain of PMMA to obtain acyl chloride groups. Next, acyl chloride groups decompose under light irradiation, introducing free radicals onto the main chain through a series of reactions. Finally, β–fracture of main chain occurs and depolymerization begins. The dependence of PMMA depolymerization on light in TCM comes from two aspects: (1) photooxidation of TCM to produce phosgene; (2) photolysis of side chain acyl chloride groups.

### 3.3. Validation of the Mechanism

**Figure 9:**
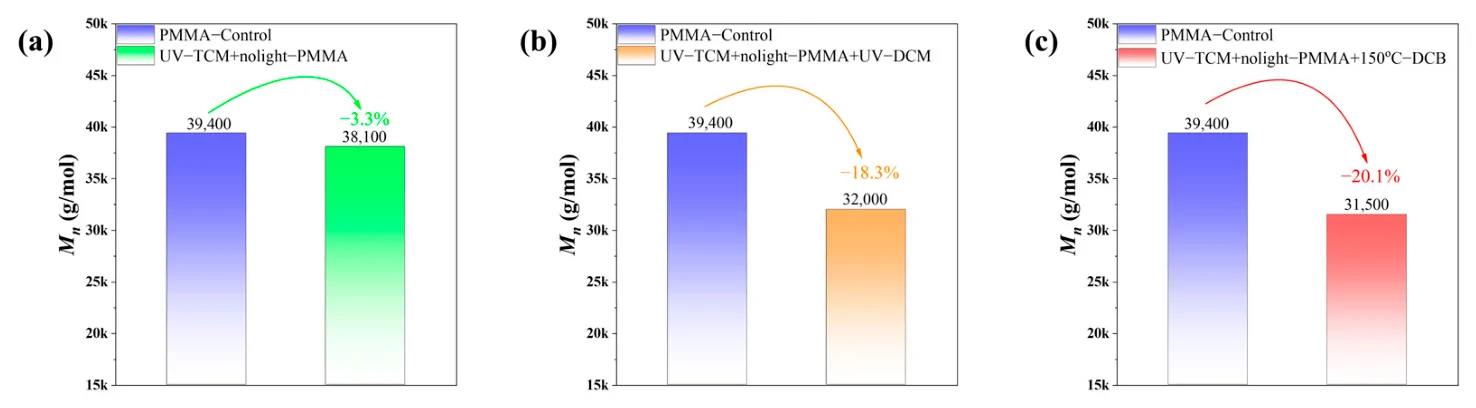
Changes in molecular weight of (**a**) UV−TCM + non light−PMMA reaction; (**b**) UV−TCM + non light−PMMA + UV−DCM reaction; (**c**) UV−TCM + non light−PMMA + 150 °C−DMB reaction.

Based on the above mechanism, both the formation of phosgene and the photolysis of side chain acyl chloride groups need the participation of light. If light is only provided for the photooxidation process of TCM, PMMA will not undergo a complete degradation process. To demonstrate this assumption, we first irradiated TCM with UV light for 12 h to accumulate phosgene, and then dissolved PMMA in dark for 24 h. Due to the lack of light required for the cleavage of side chain acyl chloride groups, the degradation of PMMA should not occur. This assumption was proved in Figure 9a. The molecular weight of PMMA only decreased by 3.3%, which means that the degradation of PMMA hardly occurred. Then the product was vacuum dried in the absence of light for 8 h to remove TCM, phosgene, and trace MMA monomers. According to Figure 8, this product should be PMMA with partially acylated side chains. Next, this product was dissolved in DCM and subjected to UV irradiation for 24 h. Figure 9b shows that the PMMA molecular weight was decreased by 18.3% after the “UV + non light + UV−DCM” process. Therefore, two stages of light participation in the chain-scission reaction of PMMA in TCM were demonstrated.

In Figure 1, the molecular weight remained nearly unchanged in dark, but an MMA monomer peak was clearly observed in Py-GC/MS test. Considering that the sample underwent a high temperature procedure during the flash evaporation analysis at 300 °C, it is suggested that the PMMA degradation may also be initiated by heat. To prove this, we dissolved the product of the “UV + non light” reaction in dimethylbenzene (DMB), refluxed at 150 °C for 24 h in dark, and determined the molecular weight changes. Figure 9c shows that the PMMA molecular weight was decreased by 20.1% after the “UV + non light + 150 °C−DMB” procedure. This demonstrated clearly that cleavage of the acyl chloride group on the side chain of PMMA can also be initiated by heat.

**Figure 10:**
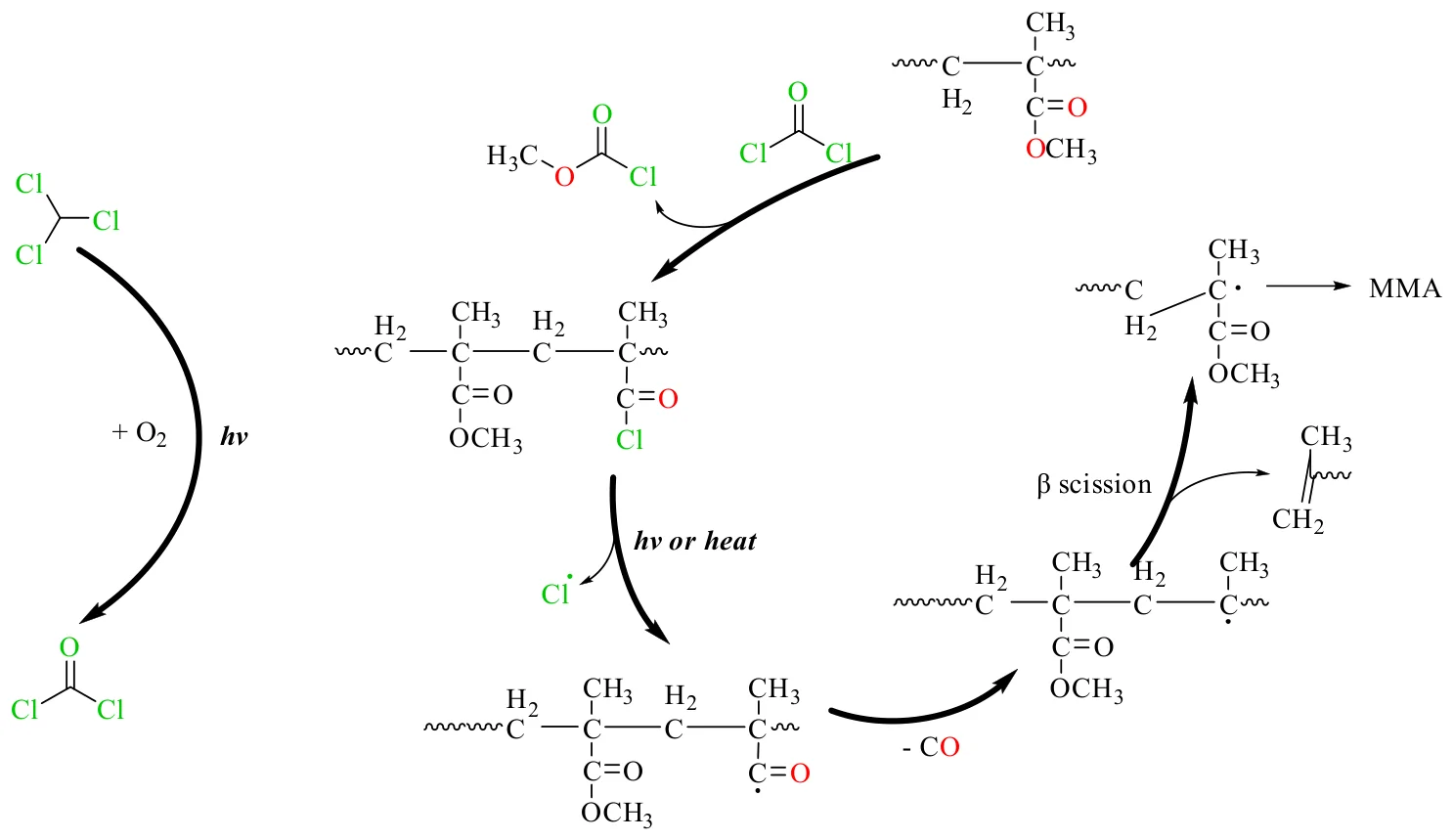
Updated side-chain-initiated main-chain scission mechanism of PMMA in TCM.

Based on the above results, an updated chain-scission mechanism of PMMA in TCM is shown in Figure 10, in which cleavage of acyl chloride groups on the side chain can be initiated by light irradiation or heat.

According to Figure 10, the amount of phosgene has great influence on the rate and efficiency of degradation. Therefore, if we adopt some methods to reduce the amount of phosgene in the system, the degradation efficiency should decrease subsequently.

**Figure 11:**
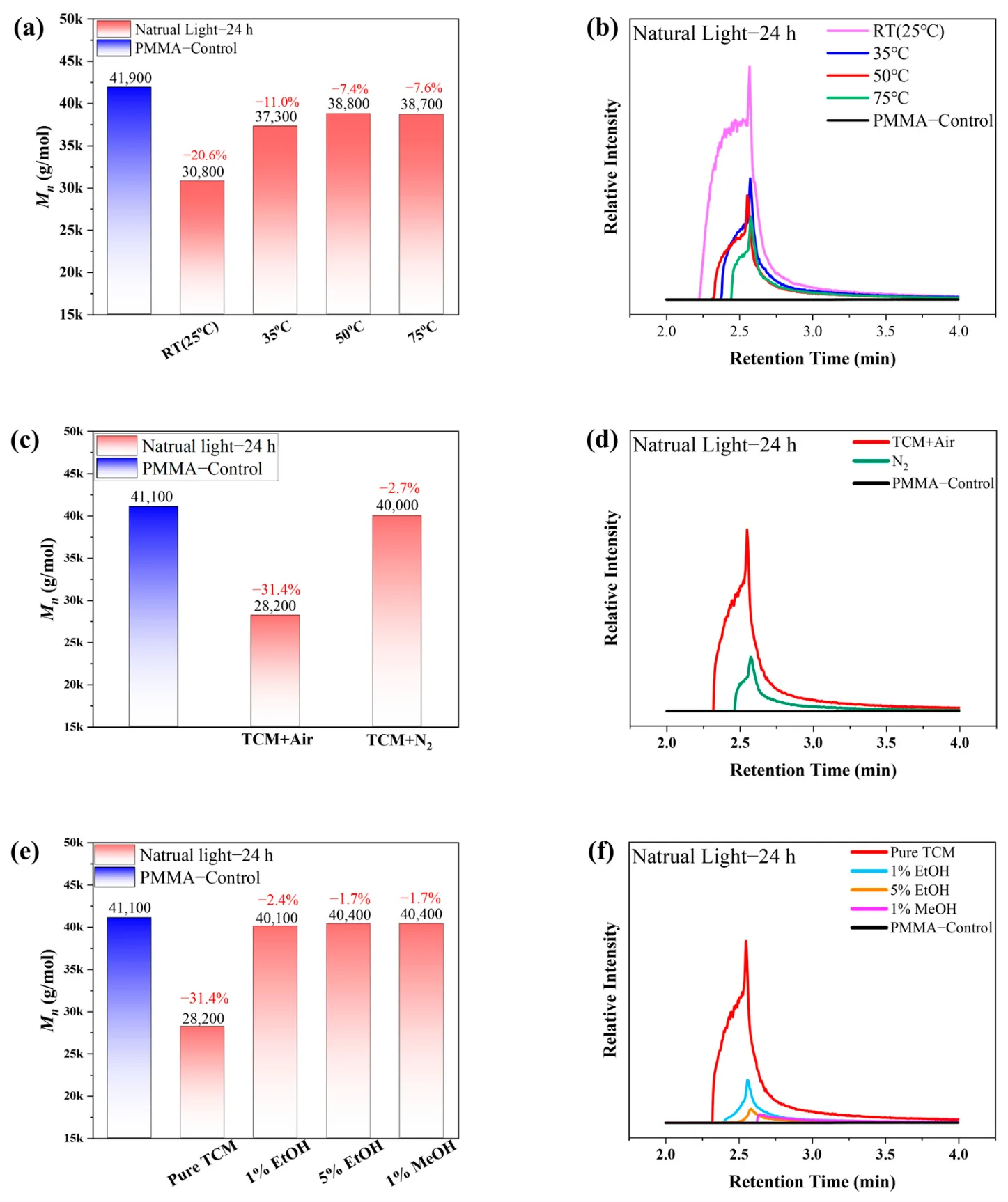
Degradation reaction of PMMA in TCM at different temperatures after 24 h: (**a**) changes in molecular weight; (**b**) peak intensity of MMA monomer (*m*/*z* = 100); degradation reaction of PMMA in TCM in N_2_ and air after 24 h; (**c**) changes in molecular weight; (**d**) peak intensity of MMA monomer (*m*/*z* = 100); degradation reaction of PMMA in TCM with different ethanol after 24 h; (**e**) changes in molecular weight; (**f**) peak intensity of MMA monomer (*m*/*z* = 100).

Due to the strong reactivity and poor stability of phosgene [29], elevated temperature will help the decomposition of phosgene and promote the escape of phosgene from the solution. As a result, the phosgene concentration in the solution will decrease and the degradation degree of PMMA will decrease accordingly. To prove this, the PMMA solution was kept under natural light for 24 h at 25 °C, 35 °C, 50 °C, and 75 °C, respectively. Figure 11a shows that as the temperature increased, the degradation degree of PMMA decreased. Figure 11b shows that the peak intensity of MMA monomer also decreased with increasing temperature. Therefore, the elevated temperature inhibited the degradation of PMMA in TCM, which was consistent with the assumption and proved the important effect of phosgene on degradation.

According to the photooxidation mechanism of TCM (Figure 2b) [27], the production of phosgene requires not only light but also the presence of oxygen. Therefore, if an anaerobic environment is set up, the production of phosgene will be suppressed, thereby inhibiting the degradation of PMMA. To prove this, we bubbled the solution with N_2_ for 5 min and sealed it to remove oxygen. Then the solution was exposed under natural light. Figure 11c shows that without oxygen, there is only a 2.7% decrease in molecular weight, much lower than the 31.4% decrease in air. Much lower MMA monomer concentration in N_2_ than in air was also detected in Figure 11d. These results clearly demonstrated that O_2_ is necessary in the production of phosgene; therefore, it is also necessary in the chain-scission reaction of PMMA.

TCM is easy to photolyze to phosgene [29]. Ethanol (EtOH) is easy to react with phosgene to obtain chloroformate and then chloroformate decomposes to obtain carbon dioxide and chlorinated alkanes [30]. Therefore, ethanol is often added as a stabilizer in TCM to remove possible phosgene [31]. Since phosgene is necessary for degradation of PMMA, if ethanol is added, phosgene will be consumed and the degradation of PMMA will be inhibited. To prove this, we added 1% and 5% ethanol, or 1% methanol to the PMMA solution respectively, and carried out the degradation under natural light. Figure 11e shows that after adding ethanol and methanol, the degradation degree of PMMA significantly decreased, and the peak intensity of MMA monomer in Figure 11f also diminished greatly. The stabilization effect of methanol and ethanol was demonstrated. And then, degradation was inhibited.

## 4. Conclusions

A photo-initiated, catalyst-free main-chain scission reaction of PMMA in TCM at room temperature was identified. The mechanism was proposed and verified based on DFT calculations and experiments. Phosgene from TCM photooxidation reacts with the ester groups on the PMMA side chain to obtain acyl chloride groups. The acyl chloride groups can be cleaved under the action of light or heat, and further trigger the main chain β-fracture. The content of phosgene in the system can be reduced by increasing the temperature, providing anaerobic conditions, and adding ethanol. Consequently, the PMMA degradation can be suppressed. This study reveals a side-chain-initiated pathway for main-chain scission of PMMA under unusually mild solution conditions. However, under these reaction conditions, PMMA mainly underwent chain scission to decrease the molecular weight, so that the yield of monomers was still relatively low. Further work is needed to quantify the product distribution in more detail and to clarify how efficiently chain scission can be controlled under different reaction conditions. In addition, understanding this mechanism may provide useful insight into both the controlled cleavage and the long-term stabilization of PMMA in chlorinated environments.

## Figures and Tables

**Table 1:** Volatile components of PMMA after 24 h under natural light irradiation in TCM.

Label	Ret. Time (min)	Name of Volatile Products	Molecular Structure
1	1.3	Carbon Dioxide	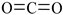
2	1.4	phosgene	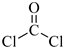
3	1.8	Trichloromethane (TCM)	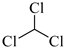
4	1.9	Tetrachloromethane	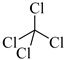
5	2.2	Trichloromethane (TCM)	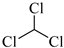
6	2.3–2.7	Methyl methacrylate (MMA)	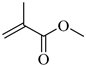

## Data Availability

The raw data supporting the conclusions of this article will be made available by the authors on request.
